# Predictors of sexual satisfaction among Social Security and National Insurance Trust pensioners in the Greater Accra Region of Ghana

**DOI:** 10.1371/journal.pone.0331747

**Published:** 2025-09-09

**Authors:** Myles Ongoh, Kwamina Abekah-Carter, Edmond A-iyeh, Mabel Oti-Boadi, Williams Agyemang-Duah

**Affiliations:** 1 LEAP Management Secretariat, Ministry of Gender, Children and Social Protection, Accra, Ghana; 2 School of Social Work, Memorial University of Newfoundland, St. John’s, Newfoundland and Labrador, Canada; 3 Agaplesion-Diakonie Klinikum, Elise-Averdieck-Straße , Rotenburg, Germany; 4 Department of Psychology, University of Ghana, Legon- Accra, Ghana; 5 Department of Public Health Sciences, Queen’s University, Kingston, Ontario, Canada; University of Professional Studies, GHANA

## Abstract

The population of pensioners remains on the rise in Ghana coupled with an intrinsic need for sexual activity and satisfaction. However, data on factors associated with sexual satisfaction among pensioners are limited in Ghana. The aim of this study was to examine the predictors of sexual satisfaction among Social Security and National Insurance Trust pensioners in the Greater Accra Region of Ghana. We employed a cross-sectional survey design in this study. Participants were recruited using cluster and stratified sampling techniques. Our analytical sample was 410 participants. Ordinal logistic regressions were employed to determine predictors of sexual satisfaction among the participants. The significance of the test was set at a p-value ≤ 0.05. The results showed that participants who were household head (AOR: 1.874, 95% CI: 1.037–3.388), who did not incur any expenditure on their household in a month (AOR: 6.290, 95% CI: 1.758–22.511) and those who undertake daily exercises were significantly (AOR: 1.981, 95% CI: 1.276–3.075) more likely to fall in one of the higher categories of sexual satisfaction compared to their counterparts. Also, the study revealed that those with secondary education (AOR:.503, 95% CI:.253-.0.999), who were in the public sector (AOR:.449, 95% CI:.237 −.850), who were very dissatisfied with health service access/use (AOR:.032, 95% CI:.002−.421) and not able to determine whether they were satisfied or dissatisfied with their health status (AOR:.518, 95% CI:.329−.816) were significantly less likely to fall in one of the higher categories of sexual satisfaction. Findings of this study suggest that household headship, education level, employment sector, expenditure on household, satisfaction with health services/use, daily exercises intake and satisfaction with health status were associated with sexual satisfaction among the participants. In relation to our findings, the implications for policy, practice and future research have been discussed for the attention of policy makers and researchers.

## Introduction

Gradually, societies across the world are experiencing a significant demographic shift, with a substantial proportion of this population transitioning into old age. By 2050, older persons aged 60 years and above will account for over two billion of the world’s population [1]. During this period, the proportion of older persons to Ghana’s total population is also expected to reach about 9.8% [2]. In Ghana, the demographic of pensioners, particularly individuals who exit the formal workforce upon reaching the designated statutory retirement age of 60 years [3], constitutes a substantial segment of this transition. Considering this development, gaining insight, and addressing the peculiar needs of the ageing population has become imperative. One crucial aspect of wellbeing, which has not gained enough attention is the sexual satisfaction among pensioners in Ghana, as well as other geographical contexts. The interplay of age and other vital socio-demographic factors in old age necessitates a detailed exploration, given its potential impact on overall life satisfaction and wellbeing.

Issues relating to sexuality and sexual satisfaction are rarely discussed openly [4,5], partly due to sociocultural considerations which largely include the perception of sex by most people especially in the developing world as an unspoken intrinsic human need [6]. In lower- and middle-Income countries (LIMCs) like Ghana, cultural taboos prevent open discussion about sexuality [7]. Older people are also generally perceived to be asexual individuals as sexual activities they engage in may not be for reproductive purposes [8,9]. These circumstances make it even more complex to openly discuss and study issues of sexual orientation or activity, as well sexual satisfaction among this population [10]. Most older people are not transparent about their sexual needs because of the stereotypes associated with their sexuality [11,12], which are often internalized [9].

Nonetheless, some extant evidence indicate that many older people actively engage in and enjoy sexual activity [4,13–17]. A study conducted in England found that 84.5% of men and 59.9% of women aged 60–69 years were sexually active [18]. In the same study, 59.3% of men and 34.3% of women aged 70–79 years, and 31.1% of men and 14.2% of women aged 80 years or older, were sexually active [18]. Further, a study conducted in Sweden reported a noticeable trend of increased sexual satisfaction, less sexual dysfunction, and more positive attitudes to sexuality in later birth cohorts in comparison with earlier birth cohorts [19]. Other studies have shown that sexual satisfaction contributes significantly to physical and psychological well-being of older adults [20–22], and improves their marital relationships [23]. Research also indicates that older persons experience both physical and psychological problems with their sexuality, which results in relationship problems with their partners [24]. These issues can adversely affect their wellbeing [25]. Consequently, overlooking their sexual needs amidst multiple comorbidities and psychological distress due to post retirement stress [26,27], and financial distress due to inadequate pension income, may create more problems for pensioners and the ageing population [28].

The predictors of sexual satisfaction are multidimensional including demographic, socio-economic, pathophysiological, and lifestyle factors [5]. For instance, in the study of Starc and colleagues [5], increasing age, lower level of education, and psychological disturbances (such as depression correlated with sexual dissatisfaction), homosexual activity, increasing number of previous sexual partners, and non-marital relationships correlated with sexual satisfaction. Although population ageing is still on the ascendancy in Ghana, and the recognition of sexual activity as a lifelong occurrence [29], not much is known about the factors that predict sexual satisfaction among older persons [10]. This gap persists despite the World Health Organization’s [30] assertion that sexuality among older persons entails more than just the sexual activity and that it also includes sex, gender identities and roles, sexual orientation, eroticism, pleasure, intimacy and reproduction. The need for extensive research on the predictors of sexual satisfaction among pensioners (over 60 years) in Ghana is extremely important especially since this area is somewhat understudied nationwide.

The present study sought to examine factors that predict sexual satisfaction among pensioners in the Greater Accra Region of Ghana. There are limited studies regarding pensioners in sub-Saharan African context including Ghana [e.g., 3], especially regarding the predictors of sexual satisfaction among this population. Moreover, there have been no previous studies on the predictors of sexual satisfaction among SSNIT pensioners in Ghana; retirees (aged 60 years and over) who are beneficiaries of Ghana’s public and most patronized pension scheme. We chose to focus on the pensioner population because these individuals experience unique challenges related to retirement [31], adjustments to new lifestyles, and changes in health [32] which can influence their sexual wellbeing compared to the broader ageing population. By focusing on this demographic, the study sought to provide insights that could inform policymakers, health officials, and pensioners themselves in designing effective interventions and support systems aimed at improving sexual satisfaction, overall well-being, and quality of life. Furthermore, it aims to contribute to a deeper understanding of the sexual wellbeing of older persons or retirees; a subject often neglected in both research and policy discussions in sub-Saharan Africa.

## Methods

### Study design and setting

This study was conducted in Ghana’s Greater Accra Region, located in the southeastern part of the country. It is bordered by the Eastern Region to the north, Volta Region to the east, Central Region to the west, and the Gulf of Guinea to the south, with a total land area of 3,245 km² [33,34]. The region was purposively selected as it has one of the highest populations of Social Security and National Insurance Trust (SSNIT) pensioners [35,36], making it a key site for research on post-retirement wellbeing. The data analyzed for this paper form part of a broader cross-sectional mixed methods project focused on the survival strategies and quality of life of SSNIT pensioners in Greater Accra (Ongoh; 2020. [unpublished]), with excerpts of this research published elsewhere [28,34,35]. The specific focus of the present study is sexual satisfaction in later life, which was addressed as a distinct thematic area within the larger project framework.

### Sampe size and sampling procedure

The study involved persons aged 60 years and above who receive pensions from the Social SSNIT. These individuals had worked in either the public or private formal sector before retiring. SSNIT was chosen for this study because it is the largest organization managing pension funds in the country and serves a wide range of retirees [35]. To determine how many participants to include, we used a standard method (Yamane’s formula) for calculating sample size [37] based on a total population of 49,673 SSNIT pensioners in the region as of 2016 [36]. This was the readily available statistics for pensioners at the time of data collection. Using a confidence level of 95% and a 5% margin of error, the estimated number of participants needed was 397. To account for people who might decline to participate or not complete the survey, we added 10% more, which brought the final target sample size to 437. A stratified sampling approach was also used to ensure that both men and women, as well as those from the public and private sectors, were fairly represented. First, we divided the total pensioner population into groups based on whether they had worked in the public or private sector. Then we further split each group by gender. This allowed for the creation of a balanced sample that reflected the makeup of the pensioner population in the region.

To reach the participants, we contacted SSNIT’s head office and received permission to conduct the study. The SSNIT research unit introduced us to a network of pensioner associations in Greater Accra. These associations were organized into smaller groups called zones, each with its own leadership. We met with the head of the main association, who connected us with the leaders of the 34 zones. Rather than receiving a list of members, which was not allowed for privacy reasons, we were invited to attend the regular meetings of these zones [28,35]. During these meetings, zone leaders introduced the researchers to the group and allowed us to explain the purpose of the research. Pensioners who were interested and available were then asked if they would like to take part. While this method did not give every pensioner an equal chance of being selected, it was the most practical and respectful option at the time. To increase fairness, we visited each meeting location up to three times, giving more people the opportunity to participate [28,35]. Recruitment and data collection took place from January 10 to August 20, 2019.

### Data Collection

Data were collected using a pre-tested structured questionnaire, administered in English via mobile devices using the Insyt electronic data collection platform. This tool offered flexibility and efficiency in capturing responses [28,34,35]. Ethical clearance was secured from the Ethics Committee for the Humanities at the University of Ghana, Legon (ECH 006/18–19). All participants gave informed consent, both verbally and in writing, prior to their participation. Participants were presumed capable of providing informed consent based on their cognitive and physical ability to engage meaningfully with the study process. After the research team had provided a clear explanation of the study’s purpose, procedures, and voluntary nature, participants were only enrolled if they demonstrated understanding and willingly agreed to participate, either in writing or verbally. No formal cognitive assessments were conducted, as participants were members of organized pensioner associations and demonstrated the capacity to consent through conversation. This procedure was reviewed and approved by the Ethics Committee.

### Variables and Measures

The outcome variable for this analysis was sexual satisfaction, measured by a single-item self-rating: “How would you rate your satisfaction with your sex life?” Responses were coded on a five-point ordinal scale ranging from 0 (very dissatisfied) to 4 (very satisfied). The predictor variables were organized into three domains. First, the demographic factors: gender (0 = male, 1 = female), religious affiliation (0 = Christian, 1 = non-Christian), age group (0 = 60–64, 1 = 65–69, 2 = 70+), marital status (0 = consensual union, 1 = married, 2 = never married/separated, 3 = widowed), household size (0 = 1–5, 1 = 6–10, 2 = above 10), head of household status (0 = no, 1 = yes), and duration of retirement (0 = < 5 years, 1 = 5–9 years, 2 = 10 + years). Second, the socio-economic factors: household expenditure (0 = < GH₵500, 1 = GH₵500–999, 2 = GH₵1000–1499, 3 = GH₵1500+), education level (0 = none, 1 = primary/JHS/middle school, 2 = secondary, 3 = vocational/technical, 4 = tertiary), employment sector (0 = public, 1 = private), occupation category (0 = administrative/managerial/clerical, 1 = civil/public service, 2 = entrepreneur/industrialist, 3 = production work, 4 = teaching, 5 = other), and monthly pension income (0 = < GH₵260, 1 = GH₵260–859, 2 = GH₵860 + , 3 = undisclosed). Lastly, the lifestyle and health-related factors: use of herbal medical services (0 = no, 1 = yes), membership in a fitness club (0 = no, 1 = yes), satisfaction with access to health services (0 = very dissatisfied to 4 = very satisfied), engagement in daily physical activity (0 = no, 1 = yes), and self-rated health status (0 = very dissatisfied to 4 = very satisfied). To assess multicollinearity among predictors, variance inflation factor (VIF) scores were computed. All variables had VIF values below 2.5, indicating acceptable levels of collinearity (see Table 1).

**Table 1:** Multi-collinearity statistics.

Variables	Tolerance	VIF
Gender	.634	1.577
Religion	.952	1.051
Age(years)	.483	2.069
Years on Retirement	.486	2.058
Marital Status	.713	1.403
Household-Size	.810	1.234
Expenditure on Dependents	.856	1.169
Household Head	.751	1.331
Education	.873	1.145
Sector of Employment	.876	1.142
Occupation	.896	1.116
Monthly Income	.867	1.154
Herbalist_	.850	1.177
Fitness Club	.830	1.205
Daily Exercises	.793	1.262
Satisfaction with Health Status	.881	1.136
Access to Health Services	.834	1.199

### Analytical approach

Descriptive statistics, including frequencies and percentages, were used to summarize sample characteristics [28,34,35]. Given the ordinal nature of the outcome variable, we employed multivariable ordinal logistic regression to identify predictors of sexual satisfaction. Three stepwise models were constructed. Model 1 included only demographic variables. Model 2 incorporated both demographic and socio-economic factors. Lastly, model 3 (final model) added lifestyle and health-related variables. The results were presented as Adjusted Odds Ratios (AORs) with corresponding 95% Confidence Intervals (CIs). Statistical significance was set at p < 0.05. Data analyses were performed using SPSS version 25.

### Inclusivity in global research

Additional information regarding the ethical, cultural, and scientific considerations specific to inclusivity in global research is included in the Supporting Information (SX Checklist).

## Results

### Sample characteristics of the participants

The sample characteristics of the participants have been reported in Table 2. In this study, we calculated a sample size of 437 participants. However, we observed missing values of 27 in some of the variables included in the analysis which were excluded from the study. Therefore, 410 participants were the analytical sample used in this study. The results indicated that 62% of the participants were identified as male and were Christians (82.4%), aged between 60–64 years (47.6%), married (70.2%) and had a household size between 1–5 individuals (54.6%). Also, 82.4% of the participants were household head, earned between GH¢500–999 (42.4%) and had been on retirement for less than 5 years (42.4%). Further, 35.6% of the participants had a primary/JHS/Middle school education, 52.7% were in the public sector and were employed in production work (25.9%). In addition, 55.1% received a monthly retirement benefit of GH¢260–859, used herbalist medical services (23.9%), joined fitness club (19.8%), satisfied with both health services access/use (25.9%) and health status (53.7%). Finally, 44.4% of the participants engaged in daily exercises and 48% were dissatisfied with their sex life (see Table 2).

**Table 2:** Sample characteristics of the participants.

Variable	Category	Frequency (N)	Percent
**Gender**	Male	254	62.0
	Female	156	38.0
**Religion**	Christian	338	82.4
	Non-Christian	72	17.6
**Age (years)**	60-64	195	47.6
	65-69	138	33.7
	70 or more	77	18.8
**Marital status**	Consensual union	10	2.4
	Married	288	70.2
	Never married/Separated	37	9.0
	Widowed	75	18.3
**Household size**	1-5	224	54.6
	6-10	153	37.3
	Above 10	33	8.0
**Household head**	Yes	338	82.4
	No	72	17.6
**Years on retirement**	Less than 5 years	174	42.4
	5-9 years	144	35.1
	10 years or more	92	22.4
**Expenditure on household (GH¢)**	None	10	2.5
	Less than 500	126	30.7
	500-999	174	42.4
	1000-1499	41	10.0
	1500 or more	59	14.4
**Education**	None	3	0.7
	Primary/JHS/Middle School	146	35.6
	Secondary	53	12.9
	Vocational/Technical	64	15.6
	Tertiary	144	35.1
**Employment sector**	Public	216	52.7
	Private	194	47.3
**Occupation**	Administrative/Managerial/Clerical	70	17.1
	Civil/Public Service	94	22.9
	Entrepreneur/Industrialist	20	4.9
	Production Work	106	25.9
	Teacher/Lecturer	77	18.8
	Other	43	10.5
**Monthly income (GH¢)**	Less than 260	47	11.5
	260-859	226	55.1
	860 or more	95	23.2
	Did not disclose	42	10.2
**Use of herbalist medical services**	Yes	98	23.9
	No	312	76.1
**Joining of fitness club**	Yes	81	19.8
	No	329	80.2
**Satisfaction with health services access/use**	very dissatisfied	4	1.0
	Dissatisfied	102	24.9
	Neutral	184	44.9
	Satisfied	106	25.9
	Very satisfied	14	3.4
**Sexual satisfaction**	very dissatisfied	24	5.9
	Dissatisfied	197	48.0
	Neutral	104	25.4
	Satisfied	69	16.8
	very satisfied	16	3.9
**Satisfaction with health status**	very dissatisfied	7	1.7
	Dissatisfied	45	11.0
	Neutral	138	33.7
	Satisfied	220	53.7
**Daily exercises**	Yes	182	44.4
	No	228	55.6

### Main regression analysis

Factors predicting sexual satisfaction among the participants are reported in Table 3. In Model 1, the analysis showed that participants who had been on retirement for between 5−9 years were significantly more likely to fall in one of the higher categories of satisfaction with their sex life compared to those who had retired 10 or more years (AOR: 2.113, 95% CI: 1.088–4.104). In Model 2, the results demonstrated that participants who had secondary education significantly had a lower likelihood of falling in one of the higher categories of sexual satisfaction compared to those had tertiary level of education (AOR:.468, 95% CI:.240−.912). The results revealed that participants who were in public sector were significantly less likely to fall in one of the lower categories of sexual satisfaction compared to those who were in the private sector (AOR:.409, 95% CI:.218 −.765).

**Table 3:** Sex satisfaction.

	Model 1			Model 2			Model 3 (Final Model)		
		95% CI for AOR			95% CI for AOR			95% CI for AOR	
DEMOGRAPHIC	AOR	Lower	Upper	AOR	Lower	Upper	AOR	Lower	Upper
***Gender***									
Male	.896	.569	1.409	.741	.460	1.194	.710	.433	1.165
Female (ref)	1.00			1.00			1.00		
***Religion***									
Christian	.788	.491	1.264	.720	.437	1.188	.683	.410	1.138
Non-Christian (ref)	1.00			1.00			1.00		
***Age (years)***									
60-64	1.446	.725	2.885	1.593	.762	3.329	1.736	.812	3.709
65-69	.555	.277	1.114	.674	.324	1.403	.726	.340	1.550
70 or more	1.00			1.00			1.00		
									
***Marital Status***									
Consensual union	.624	.161	2.416	.532	.128	2.204	.525	.122	2.255
Married	1.329	.766	2.303	1.447	.810	2.583	1.573	.862	2.870
Never married/Separated	1.460	.679	3.139	1.469	.668	3.232	1.366	.604	3.090
Widowed (ref)	1.00			1.00			1.00		
***Household Size***									
1-5	.577	.294	1.134	.508	.247	1.046	.518	.246	1.089
6-10	.876	.441	1.738	.783	.381	1.611	.660	.311	1.400
Above 10 (ref)	1.00			1.00			1.00		
***Household Head***									
Yes	1.444	.834	2.502	1.740	.981	3.087	1.874*	***1.037***	***3.388***
No (ref)	1.00			1.00			1.00		
***Number of Years on Retirement***									
Less than 5 years	1.412	.714	2.792	1.273	.627	2.586	1.308	.636	2.690
5-9 years	2.113*	***1.088***	***4.104***	1.775	.888	3.547	1.841	.906	3.740
10 years or more (ref)	1.00			1.00			1.00		
**SOCIO-ECONOMIC FACTORS**									
***Education***									
None				.370	.037	3.672	.624	.062	6.281
Primary/JHS/Middle School				.661	.400	1.092	.742	.443	1.241
Secondary				.468*	***.240***	***.912***	.503*	***.253***	***0.999***
Vocational/Technical				.723	.397	1.319	.733	.399	1.348
Tertiary (ref)							1.00		
***Employment Sector***									
Public				.409*	**.218**	**.765**	.449*	**.237**	**.850**
Private (ref)							1.00		
***Occupation***									
Administrative/Managerial/Clerical				1.203	.561	2.582	1.076	.491	2.358
Civil/Public Service				.925	.433	1.973	.764	.346	1.686
Entrepreneur/Industrialist				1.234	.430	3.542	.820	.278	2.413
Production Work				.817	.384	1.739	.776	.359	1.675
Teacher/Lecturer				2.012	.904	4.475	1.590	.699	3.619
Other				1.00			1.00		
***Monthly Income (***GH¢)**)**									
Less than 260				1.425	.617	3.294	1.386	.589	3.264
260-859				1.395	.728	2.673	1.297	.650	2.589
860 or more				1.212	.574	2.558	1.065	.493	2.299
Did not disclose their income (ref)				1.00			1.00		
***Expenditure on Household (***GH¢)									
None				3.011	.861	10.532	6.290*	***1.758***	***22.511***
Less than 500				1.300	.720	2.346	1.573	.850	2.910
500-999				1.286	.586	2.823	1.307	.583	2.933
1000-1499				1.190	.635	2.229	1.327	.692	2.543
1500 or more (ref)				1.00			1.00		
**LIFESTYLE/HEALTH-RELATED**									
***Use of Herbalist Medical Care***									
Yes							1.298	.797	2.113
No (Ref)							1.00		
***Joining of Fitness Club***									
Yes							1.307	.774	2.207
No (Ref)							1.00		
***Satisfaction with Health Services***									
very dissatisfied							.032**	***.002***	***.421***
Dissatisfied							.645	.209	1.989
Neutral							.819	.275	2.434
Satisfied							1.376	.461	4.111
Very satisfied (Ref)							1.00		
***Daily Exercises***									
Yes							1.981**	***1.276***	***3.075***
No							1.00		
***Satisfaction with Health Status***									
very dissatisfied							1.271	.297	5.447
Dissatisfied							.536	.276	1.042
Neutral							.518*	**.329**	**.816**
Satisfied (Ref)							1.00		
									

*Test is significant at the 0.05 level

**Test is significant at the 0.01 level

***Test is significant at the 0.001 level

NB: Italic values indicate significance of the test.

The final model (3), participants who were household head were significantly more likely to fall in the higher categories of sexual satisfaction compared to those who were not household head (AOR: 1.874, 95% CI: 1.037–3.388). Also, those with secondary education significantly had a lower likelihood of falling in one of the higher categories of sexual satisfaction compared to those had tertiary level of education (AOR:.503, 95% CI:.253-.0.999). The results revealed that participants who were in public sector were significantly less likely to fall in one of the lower categories of sexual satisfaction compared to those who were in the private sector (AOR:.449, 95% CI:.237 −.850). The results showed that participants did not incur any expenditure on their household in a month significantly had higher odds of falling in one of the higher categories of sexual satisfaction compared to those who incurred GH¢1500 or more (AOR: 6.290, 95% CI: 1.758–22.511). Furthermore, it was found that those who were very dissatisfied with health service access/use were significantly less likely to fall in one of the higher categories of sexual satisfaction compared to those who were very satisfied with health services access/use (AOR:.032, 95% CI:.002−.421). The analysis also suggested that participants who undertake daily exercises were significantly more likely to fall in one of the higher categories of sexual satisfaction compared to those who did not undertake daily exercises (AOR: 1.981, 95% CI: 1.276–3.075). The results indicated that participants who could not determine whether they were satisfied or dissatisfied with their health status were significantly less likely to fall in one of the higher categories of sexual satisfaction (AOR:.518, 95% CI:.329−.816).

In conclusion, as per the final model, the study revealed that household headship, education level, employment sector, expenditure on household, satisfaction with health services/use, daily exercises intake and satisfaction with health status were associated with sexual satisfaction.

## Discussion

This study provides some insights into the socio-economic determinants of sexual satisfaction among pensioners in the Greater Accra Region of Ghana. Given the scarcity of empirical information on the topic of sex and sexual satisfaction among older persons in Ghana, this study is of relevance as it addresses and supplements the existing gap in knowledge. The study found that pensioners who had retired within the 5–9-year range were significantly more inclined to be satisfied with their sex life compared to those who had retired for less than 5 years, and 10 years or more. This suggests that the period since retirement could be crucial in shaping the complex interaction between retirement and sexual wellbeing among older persons. The findings are consistent with previous studies, including a mixed-method study of 103 baby boomer retirees in Singapore [38], a large-scale longitudinal household study conducted annually since 1984 in Germany [39], and a descriptive survey of 550 retirees in Ghana’s Central Region [40] — all of which established a link between retirement adjustment and life satisfaction. Hence, there is the likelihood that pensioners who had retired in the 5–9-year range may be experiencing a period of adjustment where they have settled into their new life phase and routine, possibly leading to improved overall life satisfaction, including in the realm of sexuality.

Furthermore, retirement signifies an important change that many new retirees experience on multiple levels, influencing their living standards and overall wellbeing [41]. Therefore, it is possible that those retired for less than five years are still adjusting to retirement, with documented factors such as lifestyle, health, and relationship dynamics influencing their sexual satisfaction. For instance, a UK study using data from 3,343 persons aged 55–74 found that lifestyle factors significantly affect later-life sexual wellbeing [42]. A U.S. study in Florida using a cross-sectional mixed-mode approach [43], a multi-centre study in China involving 3,001 older persons [44], and a qualitative study involving 37 older persons in South Africa [45] all highlighted the role of health status in sexual satisfaction. Additionally, a multi-country survey of 1,009 couples from Brazil, Germany, Japan, Spain, and the U.S. identified relationship quality as key to later-life sexual satisfaction [46]. Also, findings from the longitudinal Health, Aging, and Retirement Transitions in Sweden study (n = 759) showed reduced sexual wellbeing following the onset of retirement [47]. While these plausible reasons may be valid, more clarity may be attained if longitudinal studies are conducted to establish the causal relationship between sexual satisfaction and retirement period.

Significantly, the study also observed a disparity in levels of sexual satisfaction among pensioners who had attained secondary education and those who had attained tertiary education. The former were less likely to report sexual satisfaction. This instance could be attributed to several reasons. For instance, older persons with higher education may have access to a range of knowledge resources, including information on sexual health, enabling them to maintain a satisfying sexual life. This is supported by a U.S. survey involving 1,384 adults aged 45 and older [48], and a quantitative study of 571 older persons in Korea [49], which found a positive association between higher education and sexual wellbeing. On the other hand, those with a relatively lower educational background may have fewer chances for exposure to detailed information on sexual health and activity, which could result in a decreased likelihood of attaining appreciable levels of sexual satisfaction. The identified significant discrepancy in levels of sexual satisfaction between pensioners who worked in the public sector and those in the private sector may underscore the role of organizational and work environment differences in shaping the reported levels of sexual satisfactions. It is possible that the stability and routine associated with public sector works might contribute to decreased levels of stress, allowing for a more conducive space to satisfying intimate relationships. Since this analogy may not be entirely accurate for explaining this finding, conducting research on this area to derive context-specific reasons would be beneficial.

The study also found that pensioners who were household heads were more likely to report sexual satisfaction. This finding appears to differ from some studies such as one that analyzed statistical data from Wave 3 of the National Survey of Families and Households (U.S.; 2001–2003), which found that perceived equity and fairness among older partners (n = 1,920; aged 40–70) was a significant predictor of sexual satisfaction. [50]. In many Ghanaian communities, however, the presence of household heads, who are typically males, is a revered gendered norm [51]. So, there is a possibility that the role of these household heads may be providing a sense of control and stability, fostering a supportive environment for intimate relationships, and ultimately enhanced sexual satisfaction. Nonetheless, we are of the view that additional research would be useful to explore the nuances in this relationship between household heads and sexual satisfaction among pensioners. The study also observed an association between not incurring any monthly household expenditure and higher odds of falling into the higher categories of sexual satisfaction. As reported by a longitudinal study conducted in Canada and the U.S., perceived financial burdens could contribute to distress, potentially affecting overall wellbeing and sexual satisfaction [52]. Consequently, persons incurring no household expense may be experiencing less financial stress and more relaxation, which can influence their levels of sexual satisfaction.

The study also revealed that pensioners who were not satisfied with their use or access to health service less likely to report sexual satisfaction. All healthcare systems that are committed to improving the overall health of people are supposed to have resources facilitate access to health services [53]. People who express their dissatisfaction may be encountering some impediments (e.g., financial) that hinder them from effectively addressing their health concerns. This dissatisfaction and unmet health care needs could adversely impact one’s life satisfaction [54]. This study also found an association between being unable to determine satisfaction with health status and lower likelihood of reporting sexual satisfaction. While factors contributing to this may be unclear in this study, the findings suggest that the relationship between one’s awareness of their health status and sexual satisfaction could be important in understanding how self-perception can shape the dynamics of intimate experiences. As found by a quantitative study conducted on 533 participants in Israel, it could be possible that individuals who are unsure of their health status are likely to experience stress and anxiety [55], which could have an impact on various aspect of their lives, including sexual satisfaction. The study also found that undertaking daily exercises could improve sexual satisfaction among pensioners. Physical exercise is known to enhance physical and psychological wellbeing, as shown in a UK quantitative study involving 262 participants [56] and a phenomenological study in Jamestown, Ghana, with 10 participants [57]. Thus, the holistic benefits of daily exercises are likely to contribute to the higher reported levels of sexual satisfaction among older persons. Moreover, the routine practice of daily exercise may exemplify a commitment to self-care that can positively influence various aspects of life, including sexual satisfaction. Therefore, mandated institutions should encourage pensioners to engage in regular exercises activities to help boost their sexual health.

### Strengths and limitations of the study

We noted some strengths and weaknesses of this study. In terms of the strength, this is the first study to examine factors predicting sexual satisfaction among SSNIT pensioners in Ghana, thereby contributing to the empirical literature on sexual satisfaction. This study is important as it could inform healthcare providers and policymakers about the specific factors that drive sexual satisfaction among pensioners in Ghana. Such knowledge is needed to inform the design of policies and programmes intended to improve sexual satisfaction among pensioners in Ghana. For instance, in efforts to strengthen sexual satisfaction among pensioners, the findings could help identify pensioners with certain demographic, socio-economic and lifestyle characteristics who report either low or high levels of sexual satisfaction. This, in turn, could facilitate the provision of data to policymakers, enabling the development of targeted educational programmes to support pensioners with low sexual satisfaction.

For the limitations of the study, we comment that due to the cross-sectional nature of the study, we could not draw any causal relationships between the dependent (sexual satisfaction) and independent variables (demographic, socio-economic and lifestyle/health-related factors). Due to this same reason, we suggest that future research should undertake longitudinal analysis to be able to determine the causal linkages between sexual satisfaction and other demographic, socio-economic as well as lifestyle/health-related factors. Additionally, we acknowledge that the data were collected in 2019, prior to the COVID-19 pandemic and recent economic challenges in Ghana. While the core constructs studied, such as sexual satisfaction and its social and health-related correlates, may have been shaped by relatively stable factors, we recognize that newer socio-economic dynamics may influence current experiences. It is therefore recommended for future studies to replicate this work using more recent data to assess the persistence or evolution of these findings in the post-pandemic context. Furthermore, we acknowledge that coital frequency is a commonly used correlate of sexual satisfaction. However, this was not assessed in this study as the focus was primarily on the subjective sexual satisfaction perceived by pensioners. Future studies are therefore encouraged to include coital frequency where culturally and ethically appropriate.

## Conclusion

The aim of this study was to determine factors predicting sexual satisfaction among pensioners in Ghana. The key findings of the study were that household headship, education level, employment sector, expenditure on household, satisfaction with health services/use, daily exercises intake and satisfaction with health status were associated with sexual satisfaction among the participants. These findings are important to inform the design of policies and programmes intended to improve sexual satisfaction among pensioners in Ghana.

## Abbreviations

VIF: Variance Inflation Factor
SSNIT: Social Security and National Insurance Trust
SPSS: Spatial Package for Social Sciences
AOR: Adjusted Odds Ratio
CI: Confidence Interval
GH¢: Ghana Cedis
JHS: Junior High School
